# Impact of reduced robustness settings on the necessity for proton therapy treatment plan adaptation in head-and-neck cancer patients

**DOI:** 10.1016/j.phro.2026.100963

**Published:** 2026-04-11

**Authors:** Brian Zapien-Campos, Dirk Wagenaar, Tim Franssen, Hans Verbeek, Stefan Both, Peter Dendooven

**Affiliations:** a Particle Therapy Research Center (PARTREC), Department of Radiation Oncology, University Medical Center Groningen, University of Groningen, Groningen, the Netherlands; b Department of Radiation Oncology, University Medical Center Groningen, University of Groningen, Groningen, the Netherlands

**Keywords:** Robustness settings, Head-and-neck cancer, Intensity modulated proton therapy, Adaptive proton therapy

## Abstract

**Background and purpose:**

Robust optimization mitigates the effects of range and setup uncertainties in intensity–modulated proton therapy (IMPT) through robustness settings. However, it increases the dose to organs-at-risk (OARs). This study investigated how reducing range robustness (RR) and setup robustness (SR) settings impacts plan adaptation frequency in IMPT of head-and-neck cancer (HNC) patients.

**Material and methods:**

A cohort of twenty-six HNC patients treated with IMPT were retrospectively analyzed. Original plans used RR/SR settings of 3%/3 mm. For each patient, four plans were generated with reduced robustness settings. All plans were robustly evaluated on weekly repeat computed tomography scans (rCTs) across 28 error scenarios, applying 2.7–3.2% range and 1 mm setup uncertainty. The failure rate of the voxel-wise minimum D_98%_≥94% criterion was used as a surrogate for plan adaptation frequency. Differences and equivalence in failure rates between settings were assessed using both two-proportion z-test and Two-One Side Test.

**Results:**

Fixing SR at 3 mm, each 1% reduction in RR setting reduced the mean OAR dose by 0.45  Gy (RBE) and normal tissue complication probability (NTCP) by 1.1%-points at a 3.5%-points increase in adaptation triggering rate. Implementing a RR of 3%, each 1 mm reduction in SR settings reduced the OAR dose by 1.0 Gy (RBE) and NTCP by 2.2%-points at a 7.0%-points increase in adaptation triggering rate.

**Conclusions:**

Reducing robustness margins decreased OAR dose and NTCP with a manageable increase in plan adaptation; however, larger reductions would require adaptive workflows and implementation of range uncertainty mitigation strategies before safe clinical implementation.

## Highlights

• Trade-offs between robustness margins and plan adaptation rates were quantified.
• D_98%_ target coverage was used as a surrogate for plan adaptation triggering.
• Mean OAR dose decreased 0.45 Gy per % of range and 1 Gy per mm of setup reduction.
• Plan adaptation increased 3.5% per % of range and 7% per mm of setup reduction.

## Graphical abstract

**Figure:**
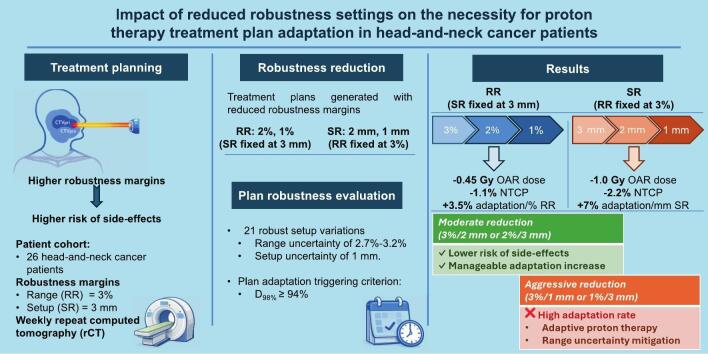

## 1 Introduction

Intensity modulated proton therapy (IMPT) has demonstrated clinical benefits over intensity modulated photon radiation therapy (IMRT) in head-and-neck cancer (HNC) patients, particularly in reducing toxicities [1], [2], [3]. The main advantage of IMPT lies in its ability to deliver highly conformal dose distributions with steep dose gradients at the Bragg peak, which more effectively spares surrounding normal tissue compared to IMRT, while ensuring adequate target coverage. However, this advantage is a double-edged sword: While IMPT minimize the dose in normal tissue adjacent to the target, it is more sensitive to treatment-related uncertainties than IMRT. This sensitivity can compromise dose conformity and lead to unintended overdosage of organs-at-risk (OARs) [4].

Treatment-related uncertainties in IMPT are categorized into range, setup and anatomical variations [5], [6]. Range uncertainties predominantly stem from inaccuracies in computed tomography (CT) calibration, i.e., the conversion of CT numbers into proton stopping power ratios (SPRs), typically introducing a systematic error of about 3% at the 1.5-standard deviation (SD) level [7], [8]. In contrast, setup uncertainties encompass inter- and intra-fractional anatomical variations, patient positioning and geometric errors, and beam reproducibility [6], [9].

To mitigate the impact of range and setup uncertainty, robust planning strategies have been employed during treatment planning to account for error scenarios that may occur during dose delivery [10], [11]. These strategies incorporate range robustness (RR) and setup robustness (SR) settings to ensure adequate target coverage throughout the treatment course, thereby enhancing treatment plan quality and reducing the risk of unintended OAR dose deviations [11], [12]. Among the available robust planning strategies, the composite minimax robust optimization (CMRO) (also known as composite worst case optimization) algorithm has been shown to enhance treatment quality compared to traditional planning target volume (PTV)-based optimization for IMPT treatment planning of HNC patients [13], [14].

Although robust planning effectively mitigates the impact of treatment-related uncertainties, there has been growing interest in evaluating whether robustness margins can be reduced to further decrease OAR dose and normal tissue complication probability (NTCP) without compromising plan quality [15], [16]. In this context, any reduction of robustness settings is conditional on the availability of independent methods to mitigate these errors.

A substantial reduction of RR and SR settings requires accurate methodologies to monitor proton beam range, ideally on a daily and online basis, to detect range and setup variations that could compromise treatment accuracy [17], [18], [19]. Current strategies addressing this need include pre-treatment or inter-fraction approaches, such as optimization of CT calibration curves by means of dual-energy CT [20], [21]. In addition, *in vivo* range verification methods capable of monitoring the range during treatment delivery are being developed. These include prompt gamma imaging [22], [23], acoustic-based methods [24], [25], and in-beam positron emission tomography (PET) [17], [26], [27], [28], which have shown promise for (near-)real-time range verification.

Recent efforts have also investigated the minimally required robustness settings to ensure adequate target coverage using a probabilistic dose accumulation method, which substantiated a 2 mm SR [16], [29]. However, this approach did not take plan adaptations into account, which could potentially compensate for anatomical changes and reduce the necessity for conservative robustness margins. At our institution, a weekly repeat CT (rCT) is acquired to evaluate whether the plan requires an adaptation based on target coverage criteria evaluated robustly across error scenarios [14]. This strategy to identify the need for weekly adaptation, together with the potential future integration of daily online adaptive protocols, increases the robustness of treatment delivery against anatomical and setup-related variations, and may reduce the need for conservative setup margins related to these effects [30], [31].

This study aimed to evaluate, using rCT-based robust plan evaluations, how variations in SR and RR settings influence the frequency of required plan adaptations to maintain adequate target coverage during HNC-IMPT treatment. Additionally, the impact of reduced robustness settings on mean OAR dose and NTCP was investigated to assess the trade-offs between the plan robustness level and plan adaptive triggering rates.

## 2 Materials and methods

### 2.1 Patient selection

A retrospective study was conducted on a cohort of 26 consecutively treated HNC patients treated with pencil beam scanning (PBS) IMPT (reviewed and approved by the Central Ethics Review Committee of the University Medical Center Groningen; Research Register No. 19746). All patients underwent a model-based selection approach based on the difference in NTCP (ΔNTCP) between IMPT and volumetric modulated arc therapy (VMAT) plans. The ΔNTCP thresholds applied for HNC patients were 10%-points for a single grade 2 complication, 15%-points for the cumulative occurrence of two grade 2 complications, or 5%-points for a single grade 3–4 complication, as outlined by the National Indication Protocols for Proton Therapy (NIPP-HNC) [32], [33]. The patient characteristics are summarized in Table1.

**Table 1:** Study population characteristics (N = 26). *One patient presented with tumors in both the oropharynx and hypopharynx sites. **Neck skin metastasis from squamous cell carcinoma. ***Treatment modalities classified according to the NTCP model for tube-feeding dependence in Wopken et al. [34] (see details in section 2.2).

**Population characteristic**	**Frequency**
**Tumor site**	
Larynx	3
Nasopharynx	7
Oral cavity	4
Oropharynx	11*
Neck**	1
**Tumor stage**	
T1-2	6
T3-4	20
**Baseline xerostomia**	
None – a bit	24
Moderate – severe	2
**Baseline dysphagia**	
Grade 0–1	17
Grade 2–3	9
**Weight loss**	
None	12
1–10%	10
>10%	4
Treatment modality***	
Chemoradiation	13
Conventional fractionation	9
Accelerated fractionation	4
**No. of rCT acquired**	
Five	3
Six	21
Seven	2
**No. of plan adaptations**	
None	21
One	4
Two	1

### 2.2 Treatment planning

For all plans, a constant proton relative biological effectiveness (RBE) factor of 1.1 was used. Patients were treated with 70 Gy (RBE)^2^ to the primary clinical target volume (CTV_pri_) and 54.25 Gy (RBE) to the prophylactic CTV (CTV_pro_). Treatment modalities are shown in Table 1. *Conventional fractionation* consisted of 35 fractions delivered over seven weeks; *accelerated fractionation* consisted of 35 fraction delivered over 6 weeks; and chemoradiation consisted of a conventional fractionation combined with a chemotherapy scheme (see details in Wopken *et al.* [34]). Patient immobilization was implemented using a 5-point thermoplastic mask (HP Pro, Orfit Industries, Wijnegem, Belgium).

Original IMPT plans were generated using the CMRO algorithm [11] within the RayStation treatment planning system (TPS) (Version 11B, RaySearch Laboratories AB, Stockholm, Sweden). The plan optimization incorporated 3% RR and 3 mm SR settings; dose calculations were computed using the Monte Carlo engine with an uncertainty of 0.5%. Robust optimization accounted for seven isocenter shifts (no shift, positive and negative shift along each axis) combined with two range shifts (positive and negative range shift). Therefore, the composite worst case over the 14 scenarios is optimized. These robustness settings reflect current clinical practice and were used as the reference configuration for all subsequent comparative analyses.

For dose calculation, a mass density-to-SPR conversion curve was used, consistent with clinical practice. CT acquisition and calibration details are described in Meijers *et al.* [20]. CT scans were acquired on a dedicated proton therapy center CT Scanner (Somatom AS Open, Siemens Healthineers, Erlangen, Germany) using the standard head-and-neck protocol with the following parameters: 120 kV, 0.98 × 0.98 mm^2^ pixel size, 2-mm thickness slice, and 300 mAs and Sinogram Affirmed Iterative Reconstruction (SAFIRE) algorithm (Siemens Healthiners, Erlangen, Germany).

Because target coverage competes with other optimization objectives (e.g., dose-volume constraints on OAR) during the plan optimization, adequate target coverage is not fully guaranteed by the optimization process. Therefore, in line with the Dutch consensus for proton plan evaluation [13], [14], after plan optimization target coverage robustness was assessed using a voxel-wise evaluation approach [14]. The robust evaluation was performed over 28 scenarios, combining 14 setup position shifts of 3 mm (positive and negative shift along each axis and diagonal) with ± 3% range uncertainty shifts. The plan was considered clinically acceptable if, in the voxel-wise minimum distribution, 98% of the CTV volume (D_98%_) received at least 94% of the prescribed dose, and if, in the voxel-wise maximum distribution, the CTV_pri_ D_2%_ remained below 110% of the prescribed dose [35].

As part of the standard IMPT workflow, weekly rCT scans were acquired to assess the need for plan adaptation due to the anatomical changes that could occur in patients during the treatment. Across the cohort, five rCTs were acquired for three patients, six for 21 patients, and seven for two patients, resulting in a total of 155 rCTs. Furthermore, four patients required one plan adaptation, and one patient required two plan adaptations during the treatment. However, for the purpose of this analysis, only the original treatment plan was used for all rCTs evaluations, even beyond fractions in which a plan adaptation was applied.

### 2.3 Adjusted treatment plans

Adjusted plans with varying range and setup robustness settings were generated through an automated process based on a voxel-wise dose-mimicking optimization approach, preserving the originally planned target coverage [16], [36]. For each patient, four adjusted plans were generated using the following RR/SR settings combinations: 2%/3 mm and 1%/3 mm, 3%/2 mm, and 3%/1 mm.

The moderate reductions (2% RR and 2 mm SR) where selected as they are considered potentially achievable in clinical practice, given the CT number-to-SPR conversion accuracy [21], [37] and patient setup uncertainties [16], [38], which are typically on the order of 1.7–2% in range and ∼ 2 mm in setup. More aggressive reductions to 1% RR and 1 mm SR were included as hypothetical scenarios for completeness, intended to assess the sensitivity of plan adaptation triggering rates under potential future reductions in range uncertainties, rather than to represent currently achievable clinical conditions.

### 2.4 Robustness evaluation and plan adaptation frequency

For each plan (original and adjusted) and patient, the treatment plan dose was recalculated on all rCTs. Robustness was evaluated across 28 error scenarios using a 1 mm position setting and a 1.5-SD range uncertainty. The 1-mm position setting accounted for delivery and intrafraction uncertainties. The 1-SD range uncertainty was previously determined by Meijers *et al.* [20] as 1.2% + 0.5 mm, corresponding to a 25% reduction relative to the theoretical range uncertainty recipe reported in the literature [8]. To express this uncertainty in a patient-specific relative form, the absolute component 0.5 mm was normalized to the monitor unit (MU)-weighted mean proton range (R_mean_) of the treatment plan. The resulting relative contribution, 0.5 mm/R_mean_, was combined with the relative term 1.2% and scaled to 1.5-SD, resulting a patient-specific range uncertainty between 2.7% and 3.2%. Voxel-wise minimum and maximum dose distributions were determined from the resulting 28-error scenarios evaluation [35].

For each treatment fraction, the dose distribution was recalculated on the rCT acquired closest in time in order to better represent the patient’s actual anatomy for that fraction. The corresponding fraction distribution were then accumulated over the full treatment course. In contrast to approaches that accumulate only nominal rCT-based dose distributions, we adopted a more conservative strategy for CTV coverage evaluation, providing a stricter assessment of target coverage over the entire treatment course. As described by Scandurra *et al. *[35], rCT-based dose recalculations were accumulated not only for the nominal distribution but also for the voxel-wise minimum and maximum distributions, yielding three accumulated dose maps.

Adequate coverage was defined when the voxel-wise minimum D_98%_ ≥ 94% criterion was satisfied, whereas dose heterogeneity (i.e., presence of hot spots) was assessed using the D_2%_ ≤ 110% criterion in the voxel-wise maximum distribution of the CTV_pri_. In our clinical practice, failure to meet these criteria, particularly the D_98%_ criterion for CTV_pri_, constitutes a trigger to consider plan adaptation.

Mean OAR doses, including the oral cavity, pharyngeal constrictor muscles (PCM; superior, medial and inferior), submandibular glands, parotid glands, and cricoid cartilage, were calculated from the accumulated nominal dose distribution.

To quantify the sensitivity of plan adaptation triggering to reduced robustness settings, the above-mentioned criteria were evaluated across all rCTs. For each combination of RR and SR settings plan, the proportion of rCTs in which the criteria were not met (failure rate) was calculated and used as a surrogate measure of the expected frequency of plan adaptation required to maintain adequate target coverage.

### 2.5 Statistical analysis

Uncertainty in failure rates was quantified using Wilson score confidence intervals (CIs) [39], which provide reliable coverage for binomial evaluations in moderate sample sizes (155 rCT evaluations).

Pairwise comparisons of failure rates between reduced and original robustness settings were performed using a two-proportion z-test [40] to determine whether the observed failure rates differed statistically between them. Two-sided p-values were adjusted by the Holm-Bonferroni correction to account for multiple testing across the comparisons between each reduced and original robustness settings, with statistical significance assessed at ⍺=0.05.

To formally evaluate clinical equivalence, a Two One-Sided Test (TOST) [41] procedure was applied using an equivalence margin (Δ) of 10%-points in failure rate, reflecting the estimated operational capacity of the clinic to accommodate additional plan adaptations, rather than a formally validated clinical threshold. Equivalence was concluded when both one-side test were significant at ⍺=0.05. All statistical analyses were performed using MATLAB (R2022b).

### 2.6 OAR dose and NTCP evaluation

Mean OAR doses were converted into NTCP for moderate-to-severe xerostomia, dysphagia grade ≥ 2, and tube feeding dependence (TFD). The NTCP values were calculated according to the validated models in NIPP-HNC version 2.2 [32].

## 3 Results

**Fig. 1:**
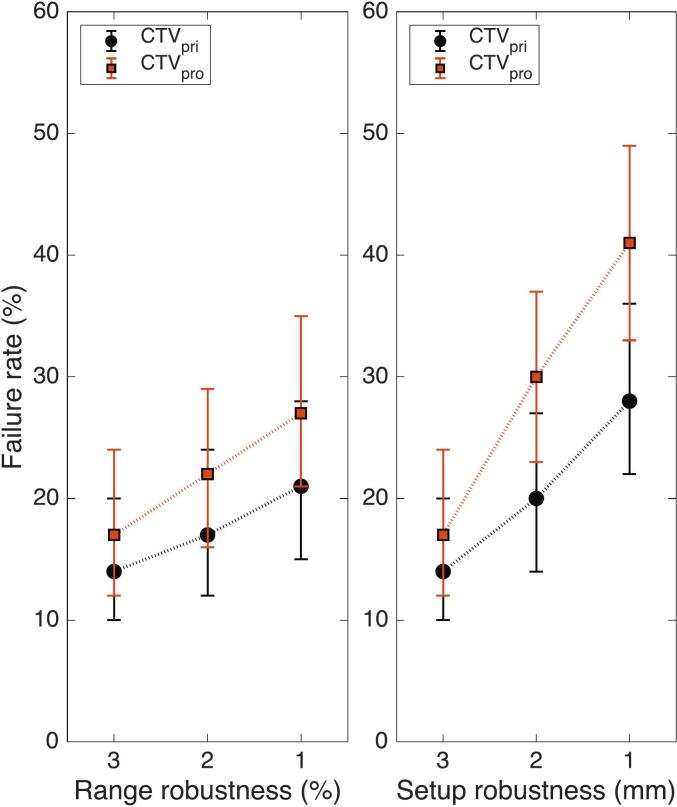
Failure rate of the voxel-wise minimum D_98%_ coverage criterion for CTV_pri_ and CTV_pro_. Failure rates are shown as a function of RR settings (left) and SR settings (right). Error bars indicate 95% Wilson score CIs.

The failure rates for the D_98%_ ≥ 94% and D_2%_ ≤ 110% criteria are summarized in Table 2. Across all robustness settings, the D_2%_ ≤ 110% criterion was satisfied, indicating consistent control of high-dose spots. Fig. 1 shows the failure rates for the D_98%_ ≥ 94% criterion.

**Table 2:** Failure rate for target dose criteria under various combinations of RR and SR settings in treatment plan optimization. Failure rates are reported for the voxel-wise minimum D_98%_ ≥ 94% criterion for both CTVs, and for voxel-wise maximum D_2%_ ≤ 110% criterion of CTV_pri_. The values are the percentage of the 155 rCTs evaluations. Wilson score CIs at 95% level are shown in brackets.

RR/SR settings	CTV_pri_ D_98%_ ≥ 94%	CTV_pro_ D_98%_ ≥ 94%	CTV_pri_ D_2%_ ≤ 110%
3%/3 mm	14% [10–20%]	17% [12–24%]	0%
2%/3 mm	17% [12–24%]	22% [16–29%]	0%
1%/3 mm	21% [15–28%]	27% [21–35%]	0%
3%/2 mm	20% [14–27%]	30% [23–37%]	0%
3%/1 mm	28% [22–36%]	41% [33–49%]	0%

For the CTV_pri_, pairwise comparisons using two-sided z-tests showed a statistically significant increase in failure rate only for the 3%/1 mm plan compared with the reference plan (p = 0.009). For the CTV_pro_, a statistically significant increase was observed for the 3%/1 mm and 3%/2 mm plans (p = 0.032 and p = 0.027, respectively). No statistically significant differences were detected for the remaining robustness settings combinations.

Equivalence testing using TOST demonstrated equivalence between the failures rates of the reference plan and the 3%/2 mm, 2%/3 mm, and 1%/3 mm plans for CTV_pri_, and between the reference plan and the 2%/3 mm plan for CTV_pro_.

**Fig. 2:**
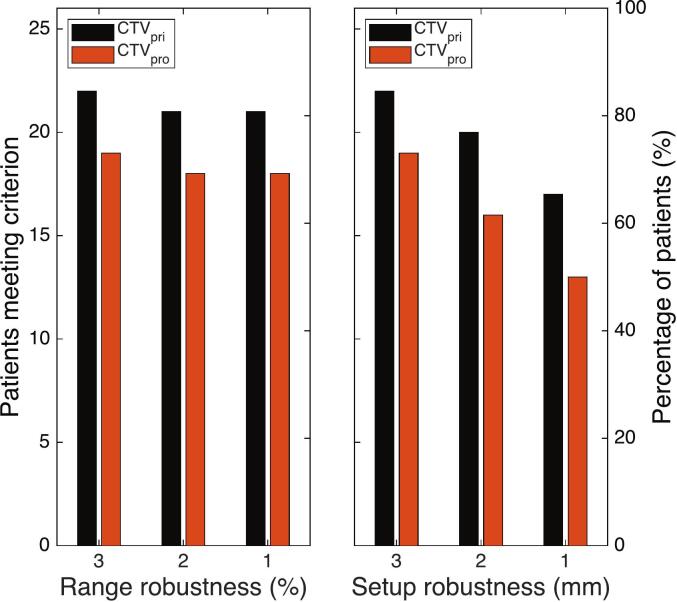
Number of patients fulfilling the D98% ≥ 94% coverage criterion for CTV_pri_ and CTV_pro_ in the accumulated voxel-wise minimum dose distribution. Left) As a function of RR setting with a fixed SR setting of 3 mm. Right) As a function of SR setting with a fixed RR setting of 3%.

Voxel-wise minimum accumulated distributions showed that for the reference plan, 22 patients met the D_98%_ coverage criterion for the CTV_pri_, and 19 patients for the CTV_pro_. Reducing RR to 2% and 1% resulted in one additional patient failing the coverage criterion for each CTV in both cases. In contrast, decreasing SR to 2 mm resulted in two and three additional failures for CTV_pri_ and CTV_pro_, respectively with respect to the reference plan. A further reduction to 1 mm SR caused five and six additional failures with respect to the reference plan for CTV_pri_ and CTV_pro_, respectively (as shown in Fig. 2).

**Fig. 3:**
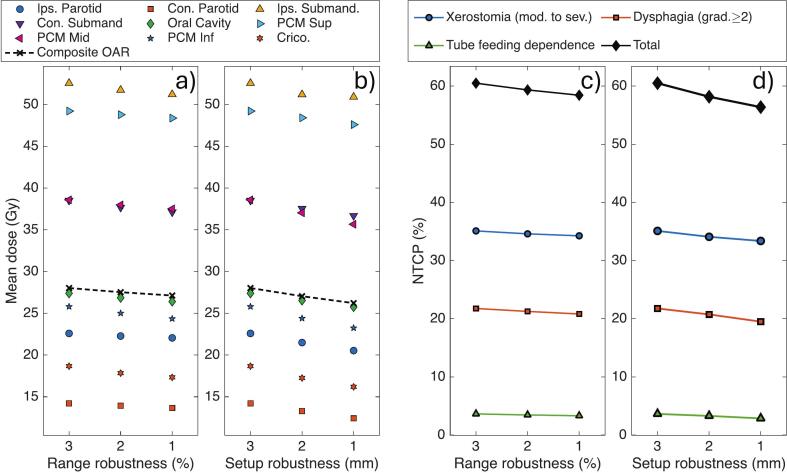
Mean OAR doses (a, b) and NTCP values (c, d) derived from the accumulated dose distributions, shown as the average over the 26 patients. Mean OAR doses are presented as function of (a) RR settings and (b) SR settings. The corresponding NTCP values for moderate-to-severe xerostomia, dysphagia grade ≥ 2, TFD, and total NTCP are shown as a function of (c) RR settings and (d) SR settings. The composite OAR was defined as the union of all other shown OARs. *Abbreviations*: Ips = Ipsilateral; Con. = Contralateral; Submand. = submandibular gland; PCM Sup/Mid/Inf = Pharyngeal constrictor muscle superior/medium/inferior; Crico = Cricopharyngeal muscle.

The mean dose of the composite OAR structure showed a linear decrease by 0.45 ± 0.01 Gy (RBE) per % RR reduction and by 1.0 ± 0.04 Gy (RBE) per mm SR reduction, as shown in Fig. 3a,b. NTCP values showed corresponding reduction of 0.4, 0.5, 0.2 and 1.1%-points per % of RR reduction and 0.8%, 1.1%, 0.3%, and 2.2%-points per mm SR reduction for moderate-to-severe xerostomia, dysphagia grade ≥ 2, TFD, and total NTCP, respectively (Fig. 3c,d). Statistical significance (p < 0.05) was observed for all endpoints except TFD with respect to RR reduction.

## 4 Discussion

This study investigated how an implementation of reduced RR and SR settings affected the frequency of weekly adaptations required during IMPT using the validated clinical surrogate criterion D_98%_ ≥ 94%. Overall, moderate reductions (2%/3 mm and 3%/2 mm) reduced OAR dose and NTCP with a clinically manageable increase in the frequency of plan adaptations.

Across all weekly rCT evaluations, the upper dose constraint for CTV_pri_ (D_2%_ ≤ 110%) was satisfied for all reduced robustness settings scenarios. For CTV_pri_, moderate reductions in SR (to 2 mm) or RR (to 1–2%) were associated with increases in adaptation frequency below 10%–points. In contrast, for the CTV_pro_, failure rates increased for the 3%/2 mm plan. However, given the lower recurrence risk associated with the CTV_pro_ [42], [43], this increase may be clinically acceptable when balanced against gains in normal tissue sparing. More aggressive SR reductions to 1 mm resulted in a substantially higher failure rate for both CTVs and, therefore, in the plan adaptation frequency.

The robustness trade-off can be summarized quantitatively. Using a SR setting fixed at 3 mm, each 1% reduction in RR yielded an average decrease of 0.45 Gy (RBE) in mean composite OAR dose and 1.1%-points in total NTCP, at the cost of an 3.5%-points increase in adaptation frequency. On the other hand, using a RR setting fixed at 3%, each 1 mm reduction in SR yielded an average decrease of 1.0 Gy (RBE) in mean composite OAR dose and 2.2%-points in total NTCP, at the cost of an 7.0%-points increase in adaptation frequency. These values illustrate cost-benefit balance between normal tissue sparing and adaptive triggering probability.

The observed OAR dose and NTCP reduction are consistent with prior work by Wagenaar *et al. *[16], who demonstrated the feasibility of 2 mm SR settings using a probabilistic dose accumulation approach. Our study extends this work by evaluating how reduced robustness settings translates into plan adaptation triggering rates. This additional endpoint directly involves departmental workload and patient throughput.

While these results support further investigation of reduced robustness margins and their potential clinical applicability in HNC-IMPT, smaller robustness settings increase both the probability and the magnitude of dose errors. Accordingly, any margin reduction should be considered conditional on reliable range uncertainty mitigation techniques, such as real-time dose/range verification methods (e.g., in-beam PET-based verification) [17], [27], and the integration of advanced image guidance within adaptive proton therapy (APT) workflows [44], [45], [46]. This strategy may enable safe exploration of reduced margins by facilitating both an early detection of clinically relevant treatment deviations and timely treatment replanning. Consistent with this, recent studies indicated that daily APT in HNC-IMPT can maintain, and in some cases improve, plan robustness with setup margins as small as 1 mm when sufficiently accurate imaging and plan adaptation strategies are in place [30], [31].

This study had some limitations, such as the cohort size, which limits the generalizability of our findings. Moreover, rCT-based evaluation does not separate systematic range errors from anatomical variations, constraining conclusions regarding reduction in RR settings. In addition, the dose accumulation approach did not account for intra-fraction random patient motion. While such effects are generally small in HNC due to rigid mask-based immobilization, positional uncertainties on the order of ∼ 1 mm may still occur due to swallowing, relaxation, or setup variations during beam delivery. Future work should model random setup errors using probabilistic approaches [16], [47] to further assess the clinical safety of reduced robustness settings.

In conclusion, this study provided evidence that moderate robustness settings reductions in IMPT robust optimization decrease OAR dose and NTCP with an acceptable increase in adaptation frequency. More substantial reductions should be pursued only alongside validated APT strategies and reliable real-time dose/range verification techniques. These findings support further investigation of robustness strategies to enhance plan quality and reduce side-effects.

## Funding statement

This work was financially supported by the Dutch Cancer Society (KWF, The Netherlands) by the RIVER project number 13240.

## CRediT authorship contribution statement

**Brian Zapien-Campos:** Writing – review & editing, Writing – original draft, Visualization, Validation, Software, Methodology, Investigation, Formal analysis, Conceptualization. **Dirk Wagenaar:** Writing – review & editing, Writing – original draft, Validation, Software, Resources, Methodology, Investigation, Data curation, Conceptualization. **Tim Franssen:** Writing – review & editing, Validation, Software. **Hans Verbeek:** Writing – review & editing, Resources, Data curation. **Stefan Both:** Writing – review & editing, Supervision, Funding acquisition, Conceptualization. **Peter Dendooven:** Writing – review & editing, Writing – original draft, Validation, Supervision, Project administration, Methodology, Investigation, Funding acquisition, Conceptualization.

## Declaration of competing interest

The authors declare that they have no known competing financial interests or personal relationships that could have appeared to influence the work reported in this paper. Given their role as a Guest Editor in Physics and Imaging in Radiation Oncology, Dirk Wagenaar had no involvement in the peer-review of this article and had no access to information regarding its peer-review. Full responsibility for the editorial process for this article was delegated to another journal editor.

## Footnotes

2 Gy (RBE) refers to the nomenclature of the International Commission on Radiation Units and Measurements (ICRU) Report 93: Prescribing, Recording, and Reporting Light Ion Beam Therapy (https://doi.org/10.1093/jicru_ndz001).
